# Environmental factors differentially affect epistaxis among preschool and school-aged children

**DOI:** 10.3389/fpubh.2023.1178531

**Published:** 2023-08-09

**Authors:** Eun-Jin Ahn, Hyun Jin Min

**Affiliations:** ^1^Department of Anesthesiology and Pain Medicine, Chung-Ang University Medical Center, Chung-Ang University College of Medicine, Seoul, Republic of Korea; ^2^Department of Otorhinolaryngology-Head and Neck Surgery, Chung-Ang University, Medical Center, Chung-Ang University College of Medicine, Seoul, Republic of Korea; ^3^Biomedical Research Institute, Chung-Ang University Hospital, Seoul, Republic of Korea

**Keywords:** epistaxis, children, environmental, differential, meteorological

## Abstract

**Introduction:**

Environmental factors are closely associated with pediatric epistaxis. Whether this association differs according to age has not been previously reported. Therefore, we tried to evaluate the differences in associations between environmental factors and epistaxis in children of different ages.

**Methods:**

A total of 20,234 patients with epistaxis who visited the hospital between January 1, 2002, and December 31, 2015, were enrolled in this study. The patients were divided into two groups according to their ages: preschool-aged (<6 years) and school-aged children (6–18 years). Daily, monthly, and yearly data on environmental factors were collected. We performed a stepwise logistic regression to identify the potential environmental risk factors for epistaxis in each age group.

**Results:**

The mean number of epistaxis cases per month in both groups was highest in September. The cases were lowest in February in preschool-aged children and in November in school-aged children. Temperature, humidity, maximum wind speed, and sunshine duration were associated with epistaxis in preschool-aged children. Average wind speed, particulate matter (>10 μm diameter), temperature, humidity, sunshine duration, and sulfur dioxide concentration were associated with epistaxis in school-aged children.

**Conclusion:**

This study indicates that the differences in environmental risk factors for epistaxis are associated with the patient’s age.

## 1. Introduction

Epistaxis is a common condition in children, with an estimated incidence of approximately 30% among children younger than 5 years and greater than 50% among children older than 5 years (1, 2). Up to 60% of all children will have had epistaxis at least once by age 10 (3). The clinical characteristics, risk factors, and management of epistaxis in children differ from those in adults. For example, unlike adult epistaxis, pediatric epistaxis most commonly originates from the anterior parts of the nose (4). In contrast to the classical dogma, which proposes that epistaxis is more common in the winter, a positive correlation has been reported between temperature and the rate of pediatric epistaxis (5). Nasal packing is the common management of epistaxis in adults. However, this procedure is insufficient for pediatric epistaxis since it may cause considerable pain and requires patient compliance, which is difficult to achieve in children (4, 6). Therefore, additional studies that focus on pediatric epistaxis and consider the unique needs of this population, which are distinct from those of adult epistaxis, are warranted.

Previous studies have described the etiology and risk factors of pediatric epistaxis. Thorsten et al. reported that infections, seasonal influences, and trauma, including nose-picking, are important etiological factors for epistaxis (4). Sophie et al. suggested that seasonal variations and patients’ sex and socioeconomic status influenced pediatric epistaxis emergency room visits (1). Recently, a study reported that air pollutants affected pediatric epistaxis, and there was a close association between the concentration of air pollutants and the incidence of pediatric epistaxis (7). In that study, the concentrations of particulate matter (PM) with diameters greater than 10 μm (PM10), PMs with diameters greater than 2.5 μm (PM2.5), sulfur dioxide (SO_2_), nitrogen dioxide (NO_2_), carbon monoxide (CO), and ozone (O_3_) were associated with pediatric epistaxis (7). However, there have been no follow-up studies regarding air pollutants and pediatric epistaxis.

In this study, we tried to evaluate the relationships between environmental factors, including meteorological factors and air pollutants, and pediatric epistaxis and to investigate whether the relationships between air pollutants and epistaxis differ between young and old children. This study utilized data from the Korea National Health Insurance Service-National Sample Cohort (NHIS-NSC), which includes a large number of patients. This study also reviewed meteorological factors and the concentration of air pollutants and compared the relationships between epistaxis in young and old children and these factors.

## 2. Materials and methods

### 2.1. Patient and public involvement

Patients and the public were not directly involved in the design, conduct, reporting, or dissemination plans of this research. This study is based on secondary analysis of anonymized data available from publicly available sources.

### 2.2. Ethical considerations and data source

This study was approved by the institutional review board of Chung-Ang University Hospital (2019–04-003), and all experiments were performed following the relevant guidelines and regulations. The requirement for written informed consent was waived by the institutional review board. The use of the database for this study was approved by the Korean National Health Insurance Service (NHIS; NHIS-2019-2-135).

The study population was selected from the NHIS-NSC, a nationwide administrative cohort created with data from the National Health Informational Database (NHID) in South Korea (8). The Korean NHIS is a single health insurer that covers approximately 97% of South Koreans and is managed by the South Korean government. The NHID is a public database that contains healthcare utilization, health screening, sociodemographic, and mortality data of the South Korean population obtained between 2002 and 2015.

### 2.3. Inclusion and exclusion criteria

**Figure 1:**
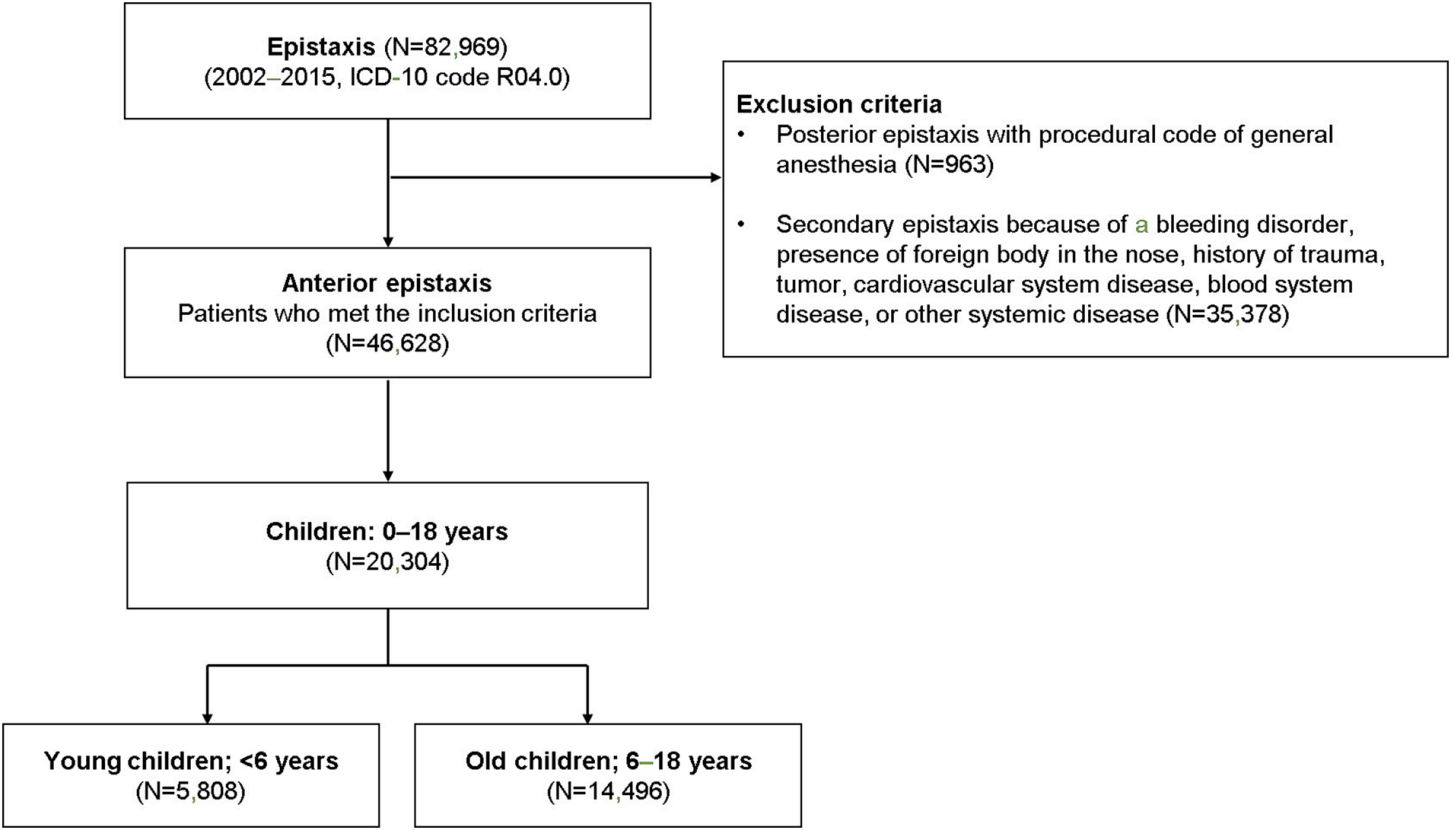
The selection of the cohort and study design. Children with potential primary epistaxis were divided into two groups based on age (preschool-aged and school-aged children).

Patients with a record of epistaxis (International Classification of Diseases [ICD]-10 code R04.0) were reviewed for this study (*n* = 82,969). Patients with suspected posterior epistaxis, defined as ICD-10 code R04.0, with the procedural code for general anesthesia (L1211 or L1212) were excluded (*n* = 963). Patients diagnosed with a nasal foreign body or with a history of trauma, tumors, cardiovascular system diseases, or other systemic diseases (ICD-10 codes C860, C833, C902, C923, C966, C722, C433, S00, S01, S02, C30, C31, C65, C66, C67, C68, D65, D66, D67, D68, S00, S01, S02, and S05) were also excluded (*n* = 35,378; Figure 1). Only children aged 0–18 years (*n* = 20,304) were included among all the eligible patients with possible anterior epistaxis.

### 2.4. Exposure variables

Data on patient characteristics, including sex, age, and hospital visit date, were obtained from the NHID. Air pollutant data, including the concentrations of PM10 (μg/m^3^), CO (ppm), O_3_ (ppm), SO_2_ (ppm), and NO_2_ (ppm), and meteorological data, including ground temperature (°C), air temperature (°C), wind speed (m/s), sunshine duration (hours), relative humidity (%), and air pressure (hPa) recorded between January 1, 2002, and December 31, 2015, were obtained from the Korea Meteorological Administration.^1^ The mean values of the data were calculated. The presence of concurrent sinonasal diseases, such as chronic sinusitis (ICD 10 code J32), acute sinusitis (ICD 10 code J01), rhinitis (ICD 10 code J31), septal deviation (ICD 10 code J342), and allergic rhinitis (ICD 10 code J30.9) was also reviewed.

### 2.5. Assessment of outcomes

The number of epistaxis cases per day, month, and year were evaluated, and they were defined as the number of first hospital visits due to epistaxis.

### 2.6. Statistical analysis

Continuous variables are presented as the mean ± standard deviation (*SD*). Stepwise linear regression models were constructed based on Akaike information Criterion (AIC) to identify the factors associated with pediatric epistaxis, and the occurrence of epistaxis was applied as the dependent variable. Among forward, backward and stepwise selection methods, stepwise selection method was applied to select and remove variables based on predefined statistical criteria. The model selection criteria were set at *p* < 0.05 for entry and *p* < 0.10 for removal of variables. At each step, variables were chosen based on the *p*-values, and a *p*-value threshold of 0.05 was used to limit the total number of variables included in the final model. All statistical tests were two-sided, and statistical significance was set at a *p* < 0.05. SAS 9.3 (SAS Institute, Cary, NC, United States) was used in all statistical analyses.

## 3. Results

**Figure 2:**
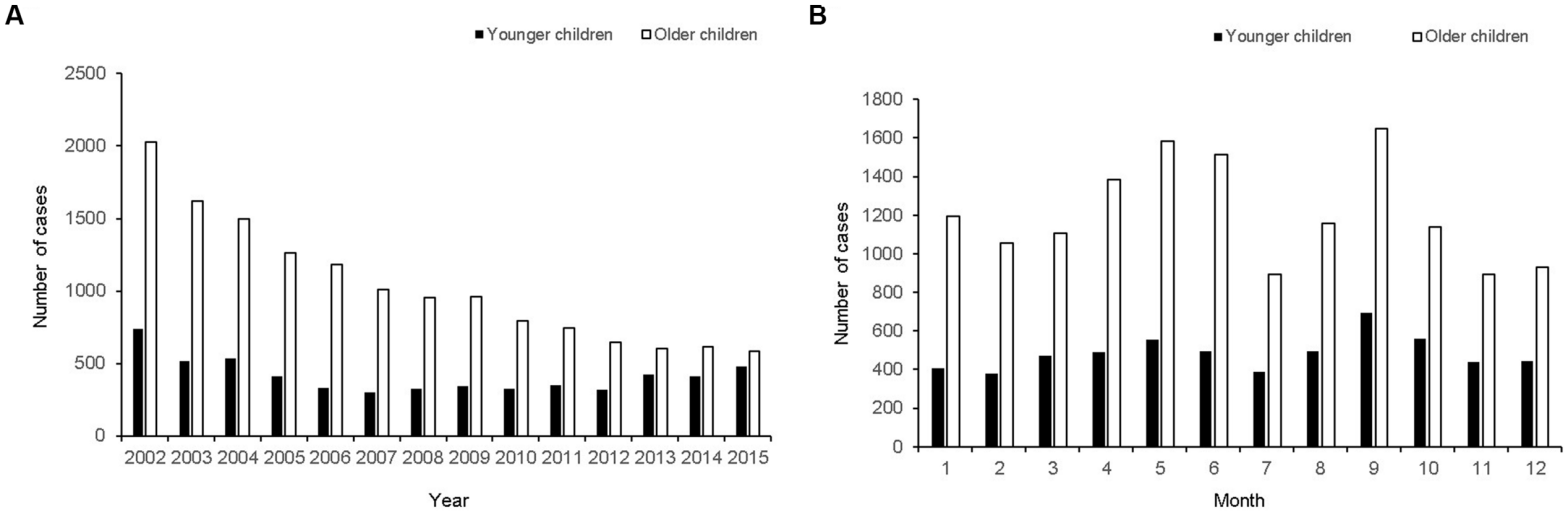
The number of epistaxis cases presented from 2002 to 2015. **(A)** Yearly distribution of potential primary epistaxis cases. **(B)** Monthly distribution of potential primary epistaxis cases.

This study included 20,304 cases (incidence rate 13.19 per 100,000) of pediatric epistaxis (Figure 1), including 5,808 cases (incidence rate 14.02 per 100,000) in young children [age: < 6 years (preschool-aged in this country), mean age: 3.5 ± 1.3 years] and 14,496 cases (incidence rate 12.89 per 100,000) in old children [age: 6–18 years (school-aged in this country), mean age: 11.0 ± 3.7 years]. The mean number of epistaxis cases per year was 414 in preschool-aged children and 1,035 in school-aged children (Figure 2A). The mean number of epistaxis cases was higher in September (11.9%) and October (9.6%) and lower in February (6.5%) and July (6.6%) in preschool-aged children (Figure 2B). In contrast, the mean was higher in September (11.4%) and May (10.9%) and lower in November (6.1%) and July (6.2%) in school-aged children (Figure 2B). South Korea’s spring season is from April to June, summer is from July to August, autumn is from September to November, and winter is from December to March.

**Figure 3:**
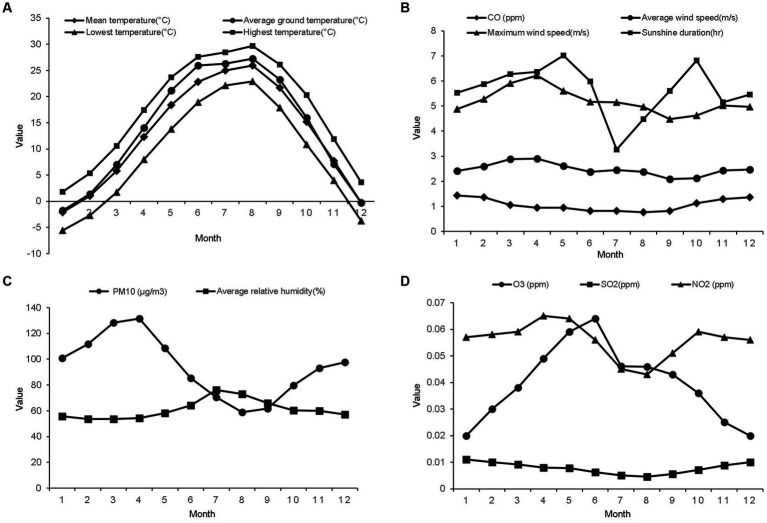
The mean values of the meteorological factors and air pollutants from 2002 to 2015. **(A)** Monthly mean values of the temperature factors. **(B)** Monthly mean values of the carbon monoxide concentration, average and maximum wind speeds, and sunshine duration. **(C)** Monthly mean values of particulate matter with a diameter of less than 10 μm and average relative humidity. **(D)** Monthly mean values of the ozone, sulfur dioxide, and nitrogen dioxide concentrations.

The temperature and relative humidity were the highest in summer and the lowest in winter (Figures 3A,C). The sunshine duration was the longest in spring and shortest in winter. The average wind speed was the highest in spring and the lowest in autumn. The mean concentration of PM10 was highest in March (131.44 ± 164 μg/m^3^) and lowest in August (58.93 ± 36 μg/m^3^). The mean concentration of CO was highest in January (1.44 ± 0.6 ppm) and lowest in September (0.82 ± 0.4 ppm). The mean concentration of O_3_ was highest in June (0.064 ± 0.03 ppm) and lowest in January (0.02 ± 0.01 ppm). The mean concentration of SO_2_ was highest in May (0.065 ± 0.02 ppm) and lowest in August (0.043 ± 0.01 ppm) (Figures 3B–D).

In the next step, we performed a stepwise regression analysis to evaluate the association between the meteorological and air pollutant factors and epistaxis in children. The average wind speed, relative humidity, PM10, sunshine duration, SO_2_, and temperature, including average ground temperature and the lowest temperature, were significantly associated with epistaxis (*p* < 0.05; Table 1).

**Table 1:** Stepwise regression model of the environmental risk factors for epistaxis in the pediatric population.

Step	Label	*p*-value	AIC	OR (95% CI)
1	Average wind speed (m/s)	<0.0001	282.07	0.65 (0.58–0.73)
2	Average relative humidity (%)	<0.0001	233.03	0.95 (0.94–0.96)
3	PM10 (ppm)	<0.0001	94.75	1.004 (1.003–1.005)
4	Sunshine duration (h)	<0.0001	52.91	0.865 (0.83–0.90)
5	SO_2_ (ppm)	<0.0001	26.66	0.995 (0.993–0.996)
6	Average ground temperature (°C)	0.0018	18.89	1.10 (1.05–1.17)
7	Lowest temperature (°C)	0.0055	11.41	0.87 (0.80–0.95)
8	Mean temperature (°C)	0.0670	10.05	1.09 (0.99–1.20)

AIC, Akaike Information Criterion; OR (95% CI), Odds ratio (95% confidence interval); PM10, particulate matter with a diameter of less than 10 μm; SO_2_, sulfur dioxide; ppm, parts per million; h, hours; m/s, meters per second.

The stepwise regression analysis, according to the age groups (preschool- vs. school-aged children), showed that the associations between the environmental factors and epistaxis were different between the two age groups. In preschool-aged children, the temperature (highest and lowest), average relative humidity, maximum wind speed, and sunshine duration were significantly associated with epistaxis (*p* < 0.05). In the school-aged children, the average wind speed, PM10, temperature (average ground temperature and lowest temperature), average relative humidity, sunshine duration, and SO_2_ were significantly associated with epistaxis (*p* < 0.05; Table 2).

**Table 2:** Stepwise regression model of the environmental risk factors for epistaxis in preschool-aged and school-aged children.

Group	Step	Label	*p*-value	AIC	OR (95%CI)
	1	Average relative humidity (%)	<0.0001	32.72	0.99 (0.98–0.99)
	2	Maximum wind speed (m/s)	0.0001	19.96	0.96 (0.93–0.98)
	3	Sunshine duration (h)	0.0172	16.27	0.98 (0.97–0.99)
Preschool-aged children	4	Lowest temperature (°C)	0.0045	10.18	0.94 (0.91–0.97)
	5	Mean temperature (°C)	0.0515	8.38	1.07 (1.04–1.11)
	6	PM10 (ppm)	0.0767	5.26	1.00 (0.99–1.00)
	1	Average wind speed (m/s)	<0.0001	282.03	0.68 (0.62–0.75)
	2	PM10 (ppm)	<0.0001	225.97	1.00 (1.00–1.00)
	3	Average ground temperature (°C)	<0.0001	181.77	1.11 (1.07–1.16)
School-aged children	4	Average relative humidity (%)	<0.0001	107.18	0.96 (0.95–0.97)
	5	Sunshine duration (h)	<0.0001	53.9	0.88 (0.85–0.91)
	6	SO_2_ (ppm)	<0.0001	17.32	0.99 (0.99–0.99)
	7	Lowest temperature (°C)	0.0015	9.25	0.93 (0.90–0.97)

AIC, Akaike Information Criterion; OR (95% CI), Odds ratio (95% confidence interval); PM10, particulate matter with a diameter of less than 10 μm; SO_2_, sulfur dioxide; ppm, parts per million; h, hours; m/s, meters per second.

Lastly, the association between nasal co-morbidities and epistaxis was evaluated. However, there were no significant associations between epistaxis and nasal co-morbidities (Supplementary Table S1).

## 4. Discussion

This study found that the association between environmental factors and epistaxis differed between preschool-aged and school-aged children. This study included patients with primary epistaxis who did not require hospitalization for surgical management. These were then divided into two groups according to their ages, and the meteorological and air pollutant factors significantly associated with epistaxis were compared between the two groups. This dataset was comprehensive and included a large, uniform study population since this study was based on data from the NHIS, which covers 97% of the country’s population and provides data collated over 14 years from one geographic area, and only included anterior epistaxis cases.

Previous studies evaluating the association between meteorological factors and epistaxis have demonstrated heterogeneous results. For example, one study failed to find a significant correlation between the incidence of epistaxis and temperature; however, another study reported that temperature was significantly associated with hospital visits for epistaxis (9, 10). Furthermore, one study reported that temperature was positively associated with epistaxis, while another reported that temperature was inversely associated with epistaxis (5, 11). These contradictory results could be attributed to the heterogeneous features of epistaxis and the age of the study population. Most existing studies regarding epistaxis are retrospective reviews of medical records, and these studies should have considered epistaxis visits to the emergency room and outpatient clinics separately.

In most cases, patients presenting to the emergency room with epistaxis have massive bleeding that requires emergent management; however, those presenting to outpatient clinics are more likely to be clinically stable. Additionally, the patient’s age should be considered since the clinical and epidemiological characteristics of epistaxis differ between children and adults (4). Furthermore, the prevalence of epistaxis requiring hospitalization differs according to age (5, 12).

Consistent with this study’s results, a previous study suggested that environmental characteristics differentially affect health parameters between young and old children (13). In that study, living in an urban environment was significantly associated with less outdoor activity and more emotional problems in old children but not young children. Therefore, the effect of environmental factors on pediatric health, especially on epistaxis, as in this study, should be differentially evaluated according to age.

Among the various air pollutants investigated, only PM10 and SO_2_ were associated with pediatric epistaxis in this study. PM is the term for a complex mixture of organic and inorganic solid particles and liquid droplets found in the air. Particles greater than 10 μm are considered more harmful since they can penetrate deeper into the lungs (14). In 2005, the World Health Organization suggested that the maximum acceptable annual average concentration of PM10 was less than or equal to 20 μg/m^3^, with a limit of less than or equal to 50 μg/m^3^ per 24-h period (15). In Korea, the annual and daily average concentrations of PM10 are less than 50 μg/m^3^ and less than 100 μg/m^3^, respectively. The Korean PM10 concentration forecast grade is designated as bad (16). Recent results from a single tertiary medical center demonstrated that the concentration of PM10 was significantly associated with epistaxis in South Korea (17). SO_2_ is typically produced from burning coal and other sulfur-containing fossil fuels. As seen in an experimental animal study, exposure to SO_2_ induces damage to the nasal mucosa (14, 18).

Consistent with the findings of this study, a previous study that included 1,330 children with a mean age of 92.5 ± 44.62 months reported that PM10 and SO_2_ levels were significantly associated with pediatric epistaxis (14). The average age of the population included in this study was relatively higher than that of the previous study (14), which might explain the similarities between this study and the previous study’s findings. Air pollutants, such as PM10 and SO_2_, stimulate immune responses in the nasal mucosa by stimulating cytokine production or inducing oxidative stress by generating reactive oxygen species (19). These pollutants should be considered significant risk factors for nasal mucosal diseases, such as epistaxis, in children since the nasal mucosa is the entry site of the pollutants, and children are more susceptible than adults to these pollutants (20).

A study from Turkey reported that the concentrations of PM10 and SO_2_ were higher in the winter and lower in the summer (14). In a study performed in Beijing, PM10 and SO_2_ were lower in the summer than in the other seasons; the PM10 concentration was the highest in early spring and autumn (7). In this study, which was performed in Korea, the PM10 concentration was the highest in March and April (spring), and the SO_2_ concentration was the highest in January and December (winter). As air pollutants show seasonal variations and are closely associated with climate variables, the effect of each air pollutant on epistaxis should be combined with the climate variables and should be differentially reported in each country. Therefore, studies that evaluate air pollutants should consider the contributions of meteorological factors. Also, this study found that environmental factors and epistaxis are differently associated between preschool-aged and school-aged children, suggesting environmental risk factors for epistaxis could be different between preschool-aged and school-aged children.

Allergic rhinitis is closely associated with epistaxis, especially in children. Pediatric patients diagnosed with allergic rhinitis are 2.4 times more likely to present with epistaxis (21, 22). However, when the association of the nasal comorbidities with epistaxis was evaluated, there was no significant association between nasal comorbidities and epistaxis (Supplementary Table S1). Further studies on this subject are needed.

This study had some limitations. First, it was based on NHIS records and did not review individual medical records. Thus, it cannot be disregarded that there is a possibility that the actual number of patients may be different from current data. Second, this study did not evaluate the delayed time effect of environmental factors on epistaxis. However, the onset of epistaxis due to air pollution does not have a noticeable delayed time effect (7). Nevertheless, as only a few studies have evaluated the delayed time effect of environmental factors on epistaxis, it is possible that environmental factors could have a delayed time effect on epistaxis. Third, this study did not consider confounding factors, such as outdoor activities and economic status. Other clinical factors such as presence of nasal infection, or allergic conditions in an individual day were not considered in current study, and our data does not suggest causal relationship. Finally, in current study, we applied a stepwise regression to manage large amounts of potential predictor variables and to choose the best predictor variables from the available options. Multicollinearity is a recognized issue in stepwise regression, where multiple predictors with high correlations are included in the regression model. The primary drawback of a stepwise regression model is its reliance on sample size. However, in this study, stepwise regression was conducted using a large sample size, which may have alleviated this problem. Nevertheless, it is important to exercise caution when interpreting the results to potential overfitting and other limitations. Future research could explore alternative variables selection techniques or validate the findings using different datasets (23).

## 5. Conclusion

In conclusion, the results of this study demonstrate that the association between environmental factors, such as meteorological factors and air pollutants, and epistaxis differs between preschool- and school-aged children. The environmental risk factors for pediatric epistaxis should be differentially evaluated according to age.

## Data availability statement

The raw data supporting the conclusions of this article will be made available by the authors, without undue reservation.

## Ethics statement

The studies involving human participants were reviewed and approved by the Chung-Ang University Hospital. Written informed consent from the participants’ legal guardian/next of kin was not required to participate in this study in accordance with the national legislation and the institutional requirements.

## Author contributions

E-JA collected the data and performed the statistical analysis. HM participated in study conceptualization, data collection, initial writing of the manuscript, and final submission. E-JA and HM reviewed the manuscript. All authors contributed to the article and approved the submitted version.

## Funding

This work was supported by the National Research Foundation of Korea (NRF) grant funded by the Korea government (MSIT) (2022R1F1A1063720). This research was also supported by research grant from Chung-Ang University Research Grants in 2022.

## Conflict of interest

The authors declare that the research was conducted in the absence of any commercial or financial relationships that could be construed as a potential conflict of interest.

## Publisher’s note

All claims expressed in this article are solely those of the authors and do not necessarily represent those of their affiliated organizations, or those of the publisher, the editors and the reviewers. Any product that may be evaluated in this article, or claim that may be made by its manufacturer, is not guaranteed or endorsed by the publisher.

## Footnotes

^1^https://web.kma.go.kr/eng/index.jsp
