# Supplementary material for: Environmental factors differentially affect epistaxis among preschool and school-aged children
Source: Front Public Health. 2023 Aug 9;11:1178531. doi: 10.3389/fpubh.2023.1178531 (PMC10446964; doi:10.3389/fpubh.2023.1178531)
Supplement: Supplementary file 1 [file Table_1.docx]

Supplementary Table 1. The prevalence of comorbidities in each age group

|  | Preschool-aged children  (*n* = 5,808) | School-aged children  *(n* = 14,496) |
| --- | --- | --- |
| Sex (male/female) | 3,135/2,673 | 5,872/8,624 |
| Age (years) | 3.5 ± 1.3 | 11 ± 3.7 |
| **Co-morbidities** |  |  |
| Allergic rhinitis | 5,723 (98.5) | 14,198 (97.9) |
| Chronic sinusitis | 3,620 (62.3) | 8,938 (61.7) |
| Acute sinusitis | 5,479 (94.3) | 12,400 (85.5) |
| Chronic rhinitis | 2,937 (50.6) | 8,624 (59.5) |
| Septal deviation | 213 (3.7) | 1,684 (11.6) |

Data are presented as the mean ± standard deviation or as a number (percentage).
